# Why does a cooled object feel heavier? Psychophysical investigations into the Weber’s Phenomenon

**DOI:** 10.1186/s12868-016-0322-3

**Published:** 2017-01-03

**Authors:** James S. Dunn, David A. Mahns, Saad S. Nagi

**Affiliations:** 1 School of Medicine, Western Sydney University, Locked Bag 1797, Penrith, NSW 2751 Australia; 2 Department of Clinical and Experimental Medicine, Center for Social and Affective Neuroscience, Linköping University, 58183 Linköping, Sweden

**Keywords:** Weber’s Phenomenon, Slowly adapting mechanoreceptor, C-tactile fibre, Heaviness perception, Touch–temperature interaction

## Abstract

**Background:**

It has long been known that a concomitantly cooled stimulus is perceived as heavier than the same object at a neutral temperature—termed *Weber’s Phenomenon* (WP). In the current study, we re-examined this phenomenon using well-controlled force and temperature stimuli to explore the complex interplay between thermal and tactile systems, and the peripheral substrates contributing to these interactions. A feedback-controlled apparatus was constructed using a mechanical stimulator attached to a 5- × 5-mm thermode. Force combinations of 0.5 and 1 N (superimposed on 1-N step) were applied to the ulnar territory of dorsal hand. One of the forces had a thermal component, being cooled from 32 to 28 °C at a rate of 2 °C/s with a 3-s static phase. The other stimulus was thermally neutral (32 °C). Participants were asked to report whether the first or the second stimulus was perceived *heavier*. These observations were obtained in the all-fibre-intact condition and following the preferential block of myelinated fibres by compression of ulnar nerve.

**Results:**

In normal condition, when the same forces were applied, all subjects displayed a clear preference for the cooled tactile stimulus as being heavier than the tactile-only stimulus. The frequency of this effect was augmented by an additional ~17% when cooling was applied concurrently with the second stimulus. Following compression block, the mean incidence of WP was significantly reduced regardless of whether cooling was applied concurrently with the first or the second stimulus. However, while the effect was abolished in case of former (elicited in <50% of trials), the compression block had little effect in four out of nine participants in case of latter who reported WP in at least 80% of trials (despite abolition of vibration and cold sensations).

**Conclusions:**

WP was found to be a robust tactile–thermal interaction in the all-fibre-intact condition. The emergence of inter-individual differences during myelinated block suggests that subjects may adopt strategies, unbeknownst to them, that focus on the dominant input (myelinated fibres, hence WP abolished by block) or the sum of convergent inputs (myelinated and C fibres, hence WP preserved during block) in order to determine differences in perceived heaviness.

**Electronic supplementary material:**

The online version of this article (doi:10.1186/s12868-016-0322-3) contains supplementary material, which is available to authorized users.

## Background

It was first reported in the mid-1800s that a cooled object is perceived as heavier than an identical object at a neutral temperature. This was first reported by Weber [1], hence called *Weber’s Phenomenon* (WP), who noted that a cold dollar coin placed on the forehead was perceived as equal, if not heavier, in weight than two warm coins placed one on top of the other. Stevens and Green [2] reinvestigated Weber’s findings and observed a robust increase in the perception of heaviness by concomitant cooling of a small weight placed on the forehead (or forearm) of subjects. Further investigations found this effect to be evident across multiple body sites, traversing both hairy and glabrous regions [3]. Interestingly, cooling the skin to 25 °C reduced the perceived magnitude of WP by an average of 9.1% [4]—a relatively minor change when compared to an earlier observation where a cooled 10-g weight was estimated to be equal in magnitude to a 100-g weight at skin temperature [2]. Investigations into tactile–heat interactions found weight intensification to be a relatively weak effect both in terms of the magnitude [2] and its distribution across body regions, only eliciting in forehead and forearm [3]. Additionally, this effect was abolished by heating or cooling the skin before the application of warmed weights [4].

It has been proposed that WP may be mediated by those mechanoreceptors that are sensitive to cooling; in particular, slowly adapting (SA) mechanoreceptors [5, 6]. While being exquisitely responsive to mechanical stimuli, about half of the SA fibres are also responsive to cooling stimuli, hence the term ‘spurious’ thermoreceptors [7–9]. Likewise, C-low threshold mechanoreceptors (C-LTMRs) are also excited by rapid cooling of the skin [10, 11]. Intriguingly, Kiesow [12] evoked a pressure sensation by evaporation of ether, a stimulus that has been shown to activate C-LTMRs [13]. However, the responsiveness of both fibre classes (i.e. C-LTMR and SA) to cooling is appreciably weaker than the activity triggered by mechanical stimulation alone or the response generated by classical cold fibres [10, 11, 14].

In the current study, we tested the production of WP using well-controlled force and cooling stimuli with the aim to better understand the complex interplay between tactile and thermal inputs. Furthermore, we tested the peripheral (myelinated vs. C fibre) contribution to this phenomenon by preferentially blocking the myelinated fibres using compression.

## Methods

Eleven healthy subjects aged 18–28 years (nine females), with no reported musculoskeletal or neurological disorders, were recruited for this study. Informed written consent was obtained from each subject before the start of the experiment. Subjects had *no* knowledge of the experimental hypothesis. This study was approved by the Human Research Ethics Committee (approval number: H9190) of the Western Sydney University in accordance with the revised Declaration of Helsinki.

Subjects sat comfortably with their right hand in a pronated position on a bench top underneath the stimulator apparatus. A single mechanical stimulator was used similar to the one in previous psychophysical studies [15–17]. Attached to the end of the stimulator was a force transducer for the accurate measurement of applied forces (ATI Force Torque Sensor, ATI Industrial Automation, North Carolina, USA). A 5- × 5-mm thermode (TSA-II Neurosensory Analyzer System, Medoc Ltd., Ramat Yishai, Israel) was attached to the test interface of the stimulator apparatus to allow precise temperature control. The dorsal surface of the hand proximal to the fifth digit was palpated to identify the boundaries of the underlying carpal bone, after which the thermode surface of the stimulator apparatus was applied to the skin overlying the bony surface with a pre-indentation force of 1 N (see Additional file 1: Fig. 1 for experiment and apparatus setup).

### Testing tactile–thermal interactions in the all-fibre-intact condition

These experiments were conducted while all nerve fibres were intact. Stimuli were presented as sequential paired forces of 0.5 or 1 N (or a combination of the two) with duration of 8-s each and an inter-stimulus interval of 5 s. Tactile-only paired stimuli were applied at the start of the experiment in order to ensure that both forces were clearly perceptible, non-painful and the subjects could easily discern between the two intensities. For test combinations, one of the paired forces was perceptibly cooled to 28 °C from a baseline of 32 °C at a rate of 2 °C/s with a 3-s static phase. At the start of the experiment, subjects were exposed to this cooling protocol to ensure that the change from 32 to 28 °C was perceptible and clearly discernible from the baseline (32 °C) temperature. The other stimulus was maintained at baseline temperature. Visual cues in the form of an automated light were provided at the onset of each stimulus as well as immediately upon cessation of the paired stimuli in order to prompt the subjects to report which of the two stimuli was perceived as heavier (based on a forced-choice paradigm) by pressing the appropriate button.

Subject responses were recorded on the same software used to drive the mechanical stimulator apparatus (Spike 2, version 6, Cambridge Electronic Design, Cambridge, England). Under control conditions (all fibres intact), there were six possible combinations of force and temperature with either the first or the second stimulus being cooled when paired forces were identical and with the smaller force being cooled when stimuli were of a different force (see Table 1). Where unequal forces were applied (at neutral temperature), the larger force was always perceived as heavier, hence cooling was not concomitantly tested with the larger force. The aim of unequal forces was to test the magnitude of WP, and hence cooling was only applied with the smaller force in the mixed-force combination. Each force-temperature combination was tested 10 times under the control condition. The order of stimulus combinations was randomised and tactile-only paired stimuli were interspersed throughout testing in order to avoid any expectation/learnt effects.

**Table 1:** Force-temperature combinations for control condition (all fibres intact)

Stimulation combinations	Force (N)		Temperature (°C)	
	Stimulus 1	Stimulus 2	Stimulus 1	Stimulus 2
1	0.5	0.5	28	32
2	1	1	28	32
3	0.5	0.5	32	28
4	1	1	32	28
5	0.5	1	28	32
6	1	0.5	32	28

### Testing tactile–thermal interactions following compression block of myelinated fibres

In a subsequent experimental sitting, the myelinated fibres of the ulnar nerve were preferentially blocked using compression of the ulnar nerve by placing a small metal slab just proximal to the medial epicondyle of humerus [16, 18]. This was successfully achieved in 9 of the 11 subjects—the block failed to take effect in one subject and another failed to show up for the experiment. The progression of the block was examined repeatedly by applying focal vibrotactile stimuli (20 Hz–20 µm; Piezo Tactile Stimulator, Dancer Design, St. Helens, UK) and thermal brass rods (~15 and ~40 °C with a contact time of 5 s) to the ulnar territory of dorsal hand. The radial territory of dorsal hand was used to compare the somatosensory sensibility across affected and intact regions. The abolition of vibration (Aβ fibres blocked) and cold (Aδ fibres blocked) sensibility was taken as indication of a functional myelinated block with the preservation of warm sensation indicative of intact C fibres [16, 19, 20].

After confirming the loss of vibration and cold sensations, test stimuli were applied to the same area of ulnar innervation as in the control experiments. However, an abridged protocol was followed in the time-constrained compression condition where only the 1-N force combinations (superimposed on a 1-N step) were tested. See stimulation combinations 2 and 4, Table 1. Both combinations were tested 10 times each.

In eight subjects, a *questionnaire*, modified from previous studies [21–23], was administered in order to assess the quality of the sensation under both control and compression conditions. See Additional file 2: Fig. 2.

### Statistical analysis

Individual subject responses and mean (±standard error of mean, SEM) data are presented as the number of times (%) the cooled stimulus was indicated to be heavier for each stimulus combination across both conditions. Two-tailed paired t tests were performed in order to detect differences based on the order of cold application. The effect of compression blockade and the order of cold application on the incidence of Weber’s Phenomenon were examined using a 2-way analysis of variance (ANOVA). Where significant differences were found (*P* < 0.05), Newman–Keuls multiple comparison tests were used to compare individual groups. Statistical analysis was performed using GraphPad Prism 6 software (GraphPad, Software, Inc., La Jolla, CA, USA).

## Results

### Weber’s Phenomenon observed reproducibly in all participants

**Fig. 1:**
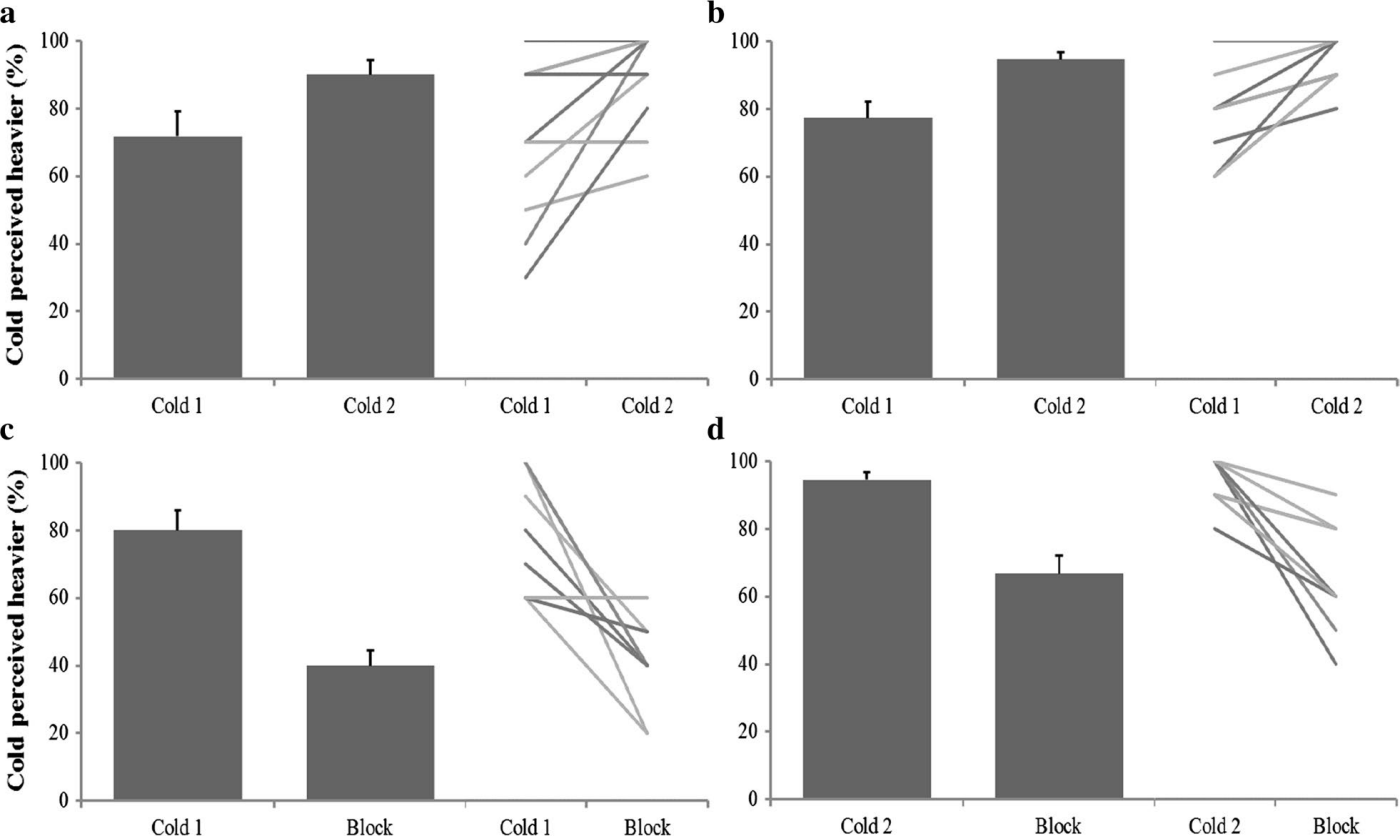
Mean (±SEM) data showing the expression of Weber’s Phenomenon in the control (all fibres intact) and compression (myelinated fibres blocked) conditions, with individual results also presented. **a**, **b** Represent control data for same-force (0.5 and 1 N, *n* = 11) combinations where Cold 1 and Cold 2 indicate whether the cooling stimulus was applied concurrently with the first or the second stimulus, **c** depicts the 1–1 N force combination where cooling was concurrent with the first stimulus before and after compression blockade of myelinated fibres (*n* = 9). Likewise, **d** represents the 1–1 N force combination before and after myelinated fibre blockade where cold was presented with the second stimulus (*n* = 9). *Lines* represent individual data of all participants, although some points may overlap

Irrespective of whether cooling was presented first or second in the control condition, all subjects (*n* = 11) consistently indicated that when the same forces were applied, the force that also had the cooling component was perceived as heavier. When paired forces of 0.5 N were applied, subjects perceived the cooled stimulus to be heavier in 71.8 ± 7.4% of trials when the first stimulus had the thermal component (Fig. 1a). When the order was reversed, subjects indicated the cooled stimulus to be heavier in 90 ± 4.3% of trials. Similar results were obtained for the 1–1 N force combinations with subjects indicating that when the cooled 1-N force was presented first it was perceived as heavier in 77.3 ± 5.6% of trials (Fig. 1b). When the cooled force was second, subjects found it to be heavier in 94.6 ± 2.1% of trials. Significant differences were revealed using paired t tests between the same-force groups (0.5–0.5 N: *P* = 0.018; 1.0–1.0 N: *P* = 0.008) based on the order of superimposed cooling, thereby suggesting an order effect within subject responses.

The mixed-force combinations comprising a cooled 0.5-N force and a thermally neutral 1-N force showed a clear preference for the latter. When the cooled 0.5-N force was presented first, it was perceived as heavier in 16.4 ± 9.0% of trials. When the cooled 0.5-N force was presented second, it was reported as heavier in 31.8 ± 11.7% of trials. The apparent order effects in mixed-force combinations were statistically indistinguishable (*P* > 0.05). Thus, while WP is quite robust when paired stimuli are of the *same* force, the effect fails to compensate for a twofold increase in force between the paired stimuli.

### Weber’s Phenomenon significantly impaired by compression block

Following the preferential block of myelinated fibres, all subjects (*n* = 9) failed to detect the 20 Hz–20 µm vibration and cooling stimuli within the ulnar innervation but readily detected warm/hot stimuli applied to the same region. In the intact condition, these participants perceived the cooled stimulus to be heavier in 80.0 ± 6.0% of trials when it was presented first, and 94.4 ± 2.7% of trials when it was presented second. Following compression, a more robust order effect (*P* < 0.001) emerged such that when cooling was applied concurrently with the first stimulus, it was perceived to be heavier in 40.0 ± 4.4% of trials, whereas, when it was applied concurrently with the second stimulus, WP was reported in 66.7 ± 5.5% of trials (Fig. 1c, d). Interestingly, in case of latter, the compression block had little effect in four out of nine participants who reported WP in at least 80% of trials (despite abolition of vibration and cold sensations). This differential effect could not have been due to an inferior compression block, as the same participants reported the abolition of WP (reported in <50% of trials) when the cooled stimulus was presented first. Newman–Keuls test revealed that the control and compression responses were significantly different (*P* < 0.001) regardless of whether cooling was presented first or second. Overall, the blockade of myelinated fibres resulted in a significant reduction in the incidence of WP (*P* < 0.001, F = 21.25), thereby suggesting a contribution of the myelinated system to this phenomenon.

### Expansion of the perceptive field following compression block

The most striking finding from the tactile questionnaire was the transition from the stimulus being described as ‘localised’ when all fibres were intact to ‘diffuse’ following compression block. Consistent with this, seven of the eight participants reported an expansion of the perceptive field, that is, the size of the skin where the percept was felt, following compression. The increase in the perceptive field—from an average size of 0.43 ± 0.04 mm in the control condition to 0.7 ± 0.05 mm in the compression condition—was statistically significant (using paired t tests, *P* = 0.001). Both control and compression experiments were indicated as non-painful by all participants. None of the participants chose the ‘cool’ descriptor for the compression condition, which is consistent with verbal reports of abolished cold sensation during nerve-block testing.

## Discussion

The current study shows that WP can be generated in a reproducible manner when stimuli of the same force are applied to the dorsal hand (hairy skin). This serves as further validation of Weber’s finding that a concomitantly cooled object can increase the sensation of pressure [1], thus making it appear as though the cooled object is heavier than the same object at neutral temperature. A number of earlier studies on WP, and indeed Weber’s own observations, used weights (or coins) with varying surface area and asked for subject’s estimation of the magnitude increase in the perceived weight of an object [1–4, 24]. With that method, at the extreme, subjects perceived a 10-g cooled weight to be equal to a 100-g weight at neutral temperature [2]. However, the magnitude of the effect size was smaller in the present study, as indicated by the preference for the 1-N tactile-only stimulus when compared to the 0.5-N cooled stimulus. This may, in part, be due to the difference in the area of activation between the earlier experiments and the current study. Whilst the 25-mm^2^ thermode allowed for localised activation within the territory of ulnar nerve, it is likely to have activated fewer receptors, which may be causative of the relatively limited effect size observed in the current study. Furthermore, the degree of thermal change is likely to be pertinent. In the current study, the temperature was only dropped from 32 to 28 °C and thus was made to be borderline perceptible whilst being clearly non-painful. This subtle temperature change is vastly different from the previous studies which immersed objects in ice water and then applied them directly to the subject’s skin, with limited capacity to account for exact temperatures or cooling rates [2–4]. The degree to which experimental design impacted on the outcomes of similar experiments is evidenced in the earlier examinations of WP in the late 70s and 80s with several papers utilising a variety of test protocols. The first foray into the re-exploration of WP revealed a remarkably large and robust intensification of touch magnitude where a 10-g cooled weight was perceived to be equal to a 100-g weight at skin temperature [2]. However, it is clear from that, and subsequent studies, that a range of factors influence the overall effect size, including temperature (both object & skin), force of stimulation/object weight, areal size of stimulation and body domain (including laterality) [2–4]. Whilst the experimental design and stimulation parameters can impact on the size of WP, the conclusive evidence from the current study and prior literature is that a cooled mechanical stimulus is perceived as heavier than a thermally neutral one, thus validating Weber’s original observations [1].

Weber’s Phenomenon was most prevalent when all fibres were intact; the sequential blockade of the myelinated afferents revealed that the remnant sense of touch following compression blockade was modulated by cooling. In the case of the Aβ-range, SA fibres are associated with the unencapsulated Merkel cells, which are located at the base of the epidermis, and the lightly encapsulated Ruffini endings, which are located in the dermis and also in joints [25, 26]. Intraneural microstimulation of single SAI afferents in the glabrous skin generates a percept of sustained pressure [23]. However, whether their counterparts in hairy skin are endowed with perceptual attributes remains unclear. As regards the SAII system, activation of individual afferents has consistently failed to evoke any sensation [23]. These SA receptors, whilst being primarily involved in the detection of mechanical events, have also been shown to respond to rapid cooling of the skin despite playing no known role in cold perception [7, 14, 19, 27]. The sensitivity of SA fibres to cooling has been taken to suggest their role in the formation of WP [6], and our stimulation parameters comprising of sustained pressure with a mild drop in temperature would seem suitable for eliciting activity in the SA fibres. Interestingly, however, a residual modulatory effect was observed during the compression block. Furthermore, the size of the perceptible field increased by >60% in the compression condition, which indicates that the residual sensation was mediated by a different class of receptors.

It is worth noting that the C cold fibres and C-LTMRs are both responsive to skin cooling, and while the former have been found to be mechanically insensitive, the latter have been implicated in ‘crude’ detection of low-force punctate mechanical stimuli [18, 28]. The residual incidence of WP following compression blockade—when the cooled stimulus was presented second (66.7 ± 5.5%)—suggests that the C-LTMRs can contribute to tactile–thermal interactions. Indeed, we have recently shown the role of C-LTMRs in thermal-pain interactions in human subjects where localised cooling of the skin overlying a painful muscle resulted in allodynia [29]. This effect was found to be independent of myelinated-fibre conduction and TRPV1 and TRPM8 function, but was abolished by the suppression of T-type calcium channel Cav3.2 that modulates the responsiveness of C-LTMRs to touch and cooling [29, 30].

While WP has primarily been hypothesised to be a result of increased discharge from a single class of afferent, namely the SA type, there is indeed the requisite circuitry at the central level that could underpin a complex perception of this kind by way of convergence and integration of inputs from multiple classes. Multi-modality interactions involving pain such as allodynia are underpinned by central convergence of inputs arising from multiple afferent classes rather than an augmented neural discharge from a single peripheral class [17, 31]. While it is evident from the current observations that changes to the peripheral drive can influence the modulatory function, whether this implicates a single class of afferent or a change in the balance of inputs from multiple classes may well reflect inter-individual differences, wherein, subjects may adopt strategies, unbeknownst to them, that focus on the dominant input (presumably SAI fibres) or the sum of convergent inputs (SA plus C fibres) in order to determine differences in perceived heaviness. As subjects make such judgements merely based on the stimulus presentation, construction of a percept dominated by SAI inputs would leave such subjects less likely to detect mechanical stimuli and WP following compression block, whereas those who built a percept based on convergent inputs may be better placed to detect WP following compression.

The apparent emergence of a priming mechanism is also noteworthy and deserving of further investigation. Across all trials, when a cold stimulus was presented second there was a much higher incidence of WP than when it was presented first. Priming mechanisms have been observed previously in response to paired tactile stimulation, for instance, where C-LTMR-optimal gentle brushing of a hairy skin site was found to have a significant impact on the level of pleasantness attributed to subsequent stimulation on the palm [32]. In that study, the role of C-LTMRs was hypothesised in the formation of this priming mechanism, a conjecture that could be extrapolated to the order effect observed in the current study. The order effect, however, appeared to be driven by the cooling component in the current study, as no such preference was observed during the same-force tactile-only trials that were interspersed among force-temperature trials. This observation, however, needs to be systematically examined in future work.

## Conclusions

In this study, we have shown that when two forces of the same magnitude are presented sequentially (in normal/all-fibre-intact condition), the cooled force is perceived as heavier than the thermally neutral one, thus validating Weber’s original observations. However, an apparent disparity in effect size was observed between the reports of previous studies and the present findings, suggesting that the experimental design is critical when examining complex perceptions. The results following compression blockade suggest that WP is most robust when all fibres are intact but that there are contributions from both the myelinated fibres and their unmyelinated counterparts.

## Additional files

**Additional file 1: Fig.** **1.** Experimental set-up with close-up of the stimulation apparatus. **A.** Subjects sat comfortably on the chair with their right hand placed in a pronated position on the surface underneath the stimulator apparatus (marked with a red rectangle). The stimulator apparatus consisted of 3 components: the mechanical stimulator, the force transducer and the 5- × 5-mm thermode attachment. The thermode was attached to the Neurosensory Analyzer system (TSA-II, not shown) that allowed precise temperature control. **B.** The stimulator apparatus in contact with the test site, overlying the cutaneous ulnar innervation.

**Additional file 2: Fig.** **2.** A copy of the questionnaire administered for assessing the quality of the sensation under both control and compression conditions.

## Abbreviations

ANOVA: analysis of variance
C-LTMR: C-low threshold mechanoreceptor
SA: slowly adapting
WP: Weber’s Phenomenon
