# Supplementary figures and images for: Why does a cooled object feel heavier? Psychophysical investigations into the Weber’s Phenomenon
Source: BMC Neurosci. 2017 Jan 3;18:4. doi: 10.1186/s12868-016-0322-3 (PMC5209941; doi:10.1186/s12868-016-0322-3)

**A.**

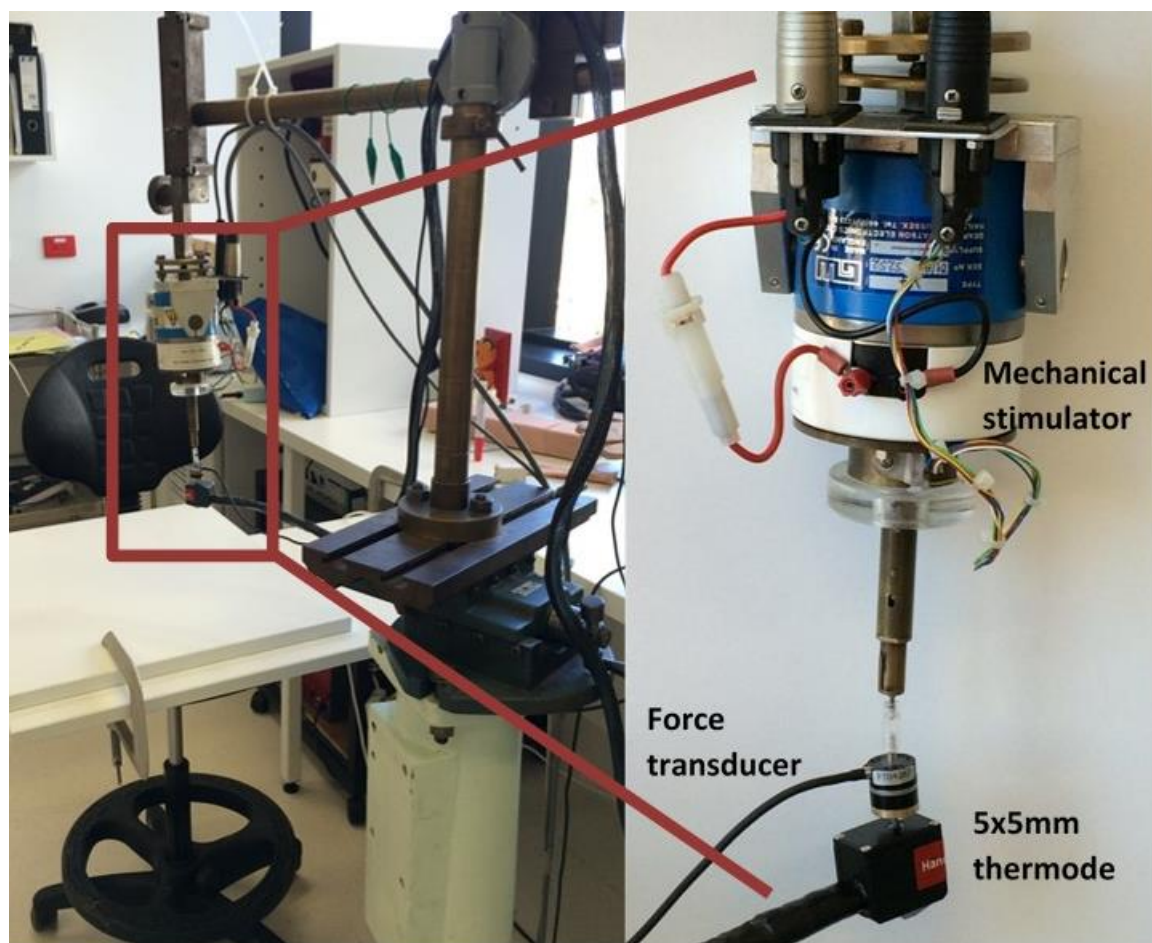

**B.**

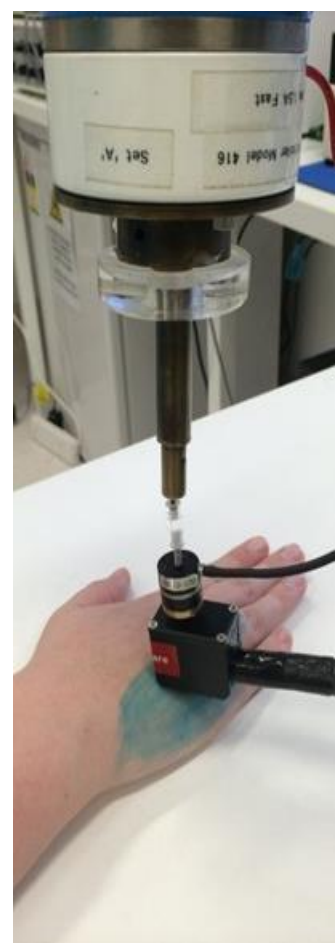

Supplement: Supplementary file 1 — Additional file 1: Fig. 1. Experimental set-up with close-up of the stimulation apparatus. A. Subjects sat comfortably on the chair with their right hand placed in a pronated position on the surface underneath the stimulator apparatus (marked with a red rectangle). The stimulator apparatus consisted of 3 components: the mechanical stimulator, the force transducer and the 5- × 5-mm thermode attachment. The thermode was attached to the Neurosensory Analyzer system (TSA-II, not shown) that allowed precise temperature control. B. The stimulator apparatus in contact with the test site, overlying the cutaneous ulnar innervation. [file 12868_2016_322_MOESM1_ESM.pdf]
