# Supplementary material for: Why does a cooled object feel heavier? Psychophysical investigations into the Weber’s Phenomenon
Source: BMC Neurosci. 2017 Jan 3;18:4. doi: 10.1186/s12868-016-0322-3 (PMC5209941; doi:10.1186/s12868-016-0322-3)
Supplement: Supplementary file 2 — Additional file 2: Fig. 2. A copy of the questionnaire administered for assessing the quality of the sensation under both control and compression conditions. [file 12868_2016_322_MOESM2_ESM.pdf]

Name:	Date:	Site of stimulation:

Category **I**: localised, diffuse;

Category **II**: instantaneous, delayed;

Category **III**: painful, non-painful;

Category **IV**: natural, unnatural (in the sense that you can imagine a real life stimulus / event which would produce the same sensation)

Category **V** *(you can choose more than one descriptor)*: light touch, pressure, stretch, traction, torsion, tickle, itch, cool, cold-pain, warm, heat-pain, burning-pain, any other kind of sensation (please describe) ______________

Category **VI**: Size of the skin area where the percept was present

Stimulus Contact Area (5-mm diameter) 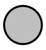

Pick the appropriate circle from the following alternatives:

Less than the smallest one <<

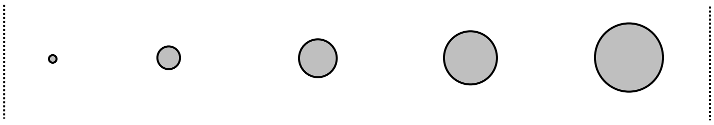

<< Greater than the biggest one

Modified from [1-3]

1. Ochoa JL, Torebjörk HE. Sensation evoked by intraneural microstimulation of single mechanoreceptor units innervating the human hand. J Physiol. 1983;342:633-654.
2. Vallbo Å, Olsson KA, Westberg KG, Clark FJ. Microstimulation of single tactile afferents from the human hand. Sensory attributes related to unit type and properties of receptive fields. Brain. 1984;107:727-749.
3. Macefield G, Gandevia SC, Burke D. Perceptual responses to microstimulation of single afferents innervating joints, muscle and skin of the human hand. J Physiol. 1990;429:113-129.
